# AFM Characterization of the Internal Mammary Artery as a Novel Target for Arterial Stiffening

**DOI:** 10.1155/2018/6340425

**Published:** 2018-11-05

**Authors:** Zhuo Chang, Paolo Paoletti, Maria Lyck Hansen, Hans Christian Beck, Po-Yu Chen, Lars Melholt Rasmussen, Riaz Akhtar

**Affiliations:** ^1^Department of Mechanical, Materials and Aerospace Engineering, School of Engineering, University of Liverpool, L69 3GH, UK; ^2^Department of Materials Science and Engineering, National Tsing Hua University, Hsinchu 30013, Taiwan; ^3^Department of Clinical Biochemistry and Pharmacology, Center for Individualized Medicine in Arterial Diseases, Odense University Hospital, University of Southern Denmark, Denmark

## Abstract

Using the atomic force microscopy- (AFM-) PeakForce quantitative nanomechanical mapping (QNM) technique, we have previously shown that the adventitia of the human internal mammary artery (IMA), tested under dehydrated conditions, is altered in patients with a high degree of arterial stiffening. In this study, we explored the nanoscale elastic modulus of the tunica media of the IMA in hydrated and dehydrated conditions from the patients with low and high arterial stiffening, as assessed *in vivo* by carotid-femoral pulse wave velocity (PWV). In both hydrated and dehydrated conditions, the medial layer was significantly stiffer in the high PWV group. The elastic modulus of the hydrated and dehydrated tunica media was significantly correlated with PWV. In the hydrated condition, the expression activity of certain small leucine-rich repeat proteoglycans (SLRPs), which are associated with arterial stiffening, were found to be negatively correlated to the medial elastic modulus. We also compared the data with our previous work on the IMA adventitia. We found that the hydrated media and dehydrated adventitia are both suitable for reflecting the development of arterial stiffening and SLRP expression. This comprehensive study of the nanomechanical properties integrated with the proteomic analysis in the IMAs demonstrates the possibility of linking structural properties and function in small biological samples with novel AFM methods. The IMA is a suitable target for predicting arterial stiffening.

## 1. Introduction

In clinical practice, arterial stiffening is commonly assessed with pulse wave velocity (PWV). PWV measurements are made *in vivo* by recording the transit time of blood across two points in the vascular system, typically from the carotid to the femoral artery. PWV is considered a powerful predictor of risk of morbidity and mortality in a general population [1, 2]. However, arteries are associated with complex structural and biomechanical processes that occur as arteries stiffen with age and disease. These alterations are not captured with PWV which simply averages the properties of the vasculature across a relatively large distance. High spatial resolution techniques such as atomic force microscopy (AFM) can bridge the gap in our understanding by enabling localized biomechanical and ultrastructural measurements on isolated sections of vascular tissue.

A number of studies have utilized high spatial resolution techniques to probe arterial stiffening *in vitro*, e.g., [3, 4]. However, these studies are generally restricted to animal models due to obvious challenges in conducting measurements on human aortic tissues. Hence, a direct link between clinical PWV and *in vitro* micro-/nanomechanical properties has long remained a challenge. Recently, the internal mammary artery (IMA) has emerged as an excellent vessel for studying the pathogenesis of arterial stiffening, especially at the molecular level [5–9]. It is readily accessible as the repair artery during coronary artery bypass graft (CABG) operations. Although it is not involved in the carotid-femoral PWV pathway, we have recently shown using AFM that alterations in nanomechanical properties and collagen fibril morphology in the IMA adventitia are strongly correlated with high PWV [1]. Furthermore, the IMA has been used in a parallel study to identify small leucine-rich proteoglycans (SLRPs) which are associated with arterial stiffening [9]. Thus, the IMA can be used to directly measure relevant properties at the ultrastructural and molecular level in patients with high PWV, overcoming the impracticability of obtaining vascular tissue from the PWV pathway.

Although the PeakForce QNM has been recently used to probe biological samples under physiological conditions [10–14], our previous study was the first to apply PeakForce QNM to vascular tissues [1]. This paper builds on that study [1] which demonstrated with PeakForce QNM AFM [15, 16] that the adventitia of the IMA is altered in patients with high PWV, in terms of both the nanoscale elastic modulus and the collagen fibril morphology. We now extend the approach and apply PeakForce QNM to the medial layer of the IMA in both dehydrated and hydrated conditions. To the best of our knowledge, this is the first study to apply the PeakForce QNM for soft tissue nanomechanical characterization in fully hydrated conditions. The paper compares the nanoscale elastic modulus of the medial layer in dehydrated and hydrated conditions, and for both conditions, the relation between this AFM data, patient metadata and proteomics data is explored. Furthermore, the dehydrated medial measurements are compared with the dehydrated adventitia measurements to determine the best approach when utilizing the IMA as an arterial stiffening target with AFM. The literature on the layer-specific biomechanical properties of the human artery is sparse, and thus this gap is addressed.

## 2. Materials and Methods

### 2.1. Patient Information

The IMA is the repair artery for CABG operations. In this study, all of the IMAs were collected from 17 patients as waste materials after the CABG operation at the Centre of Individualized Medicine in Arterial Diseases (Odense University Hospital, Odense, Denmark), as part of a project approved by the Local Ethical Committee in Region Southern Denmark (S-2010044).

Before the coronary artery bypass graft, patients were assessed by carotid-femoral PWV by using the SphygmoCor system under standardized conditions as previously described in [9]. Clinical data, including age, gender, diabetes, and hypertension, were collected before the surgery. Based on the reference and normal values for carotid-femoral PWV [17], the 17 patients were split into two groups: low PWV (8.5 ± 0.7 ms^−1^, *n* = 8 patients) and high PWV (13.4 ± 3.0 ms^−1^, *n* = 9 patients). Full patient clinical parameters are presented in [1]. It should be noted that the tissue samples and patient cohorts are identical to those in our previous paper which focussed on the IMA adventitia [1]. However, the testing location (medial layer) and methodology (dehydrated and hydrated) are different.

### 2.2. PeakForce QNM in Ambient Conditions

AFM testing was conducted on 5 *μ*m cryosections with the PeakForce QNM method using a MultiMode 8 Atomic Force Microscopy (AFM) (NanoScope VIII MultiMode AFM, Bruker Nano Inc., Nano Surface Division, Santa Barbara, CA). The method has been explained in detail elsewhere [1]. All measurements were conducted with Bruker RTESPA-150 etched silicon probes. These probes have a nominal radius of 8 nm and a cantilever with a nominal spring constant of 5 Nm^−1^ and resonant frequency of 150 kHz. The spring constant and tip radius of the probe were calibrated before measurements. In addition, a photostress coating polymer reference sample (PS1, Vishay Precision Group, Heilbronn, Germany) with known elastic modulus was used to calibrate the mechanical measurements [1].

**Figure 1:**
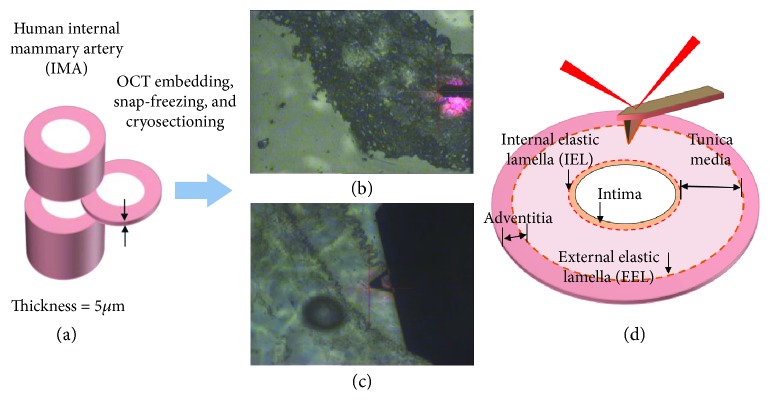
The approach for localized nanomechanical mapping of the tunica media in human IMA. (a) The human internal mammary artery (IMA) was cryo-sectioned to 5 *μ*m thickness tissue sections. Optical images captured with the microscope integrated with the AFM for the (b) dehydrated and (c) hydrated tissue sections. The IEL and EEL distinctly separate the tunica intima, tunica media, and tunica adventitia in the IMA. (d) Schematic diagram of AFM imaging and the IMA histology.

For these measurements, the scan rate was fixed at 0.93 Hz, and the resolution was set at 256 pixels per line with fixed scan size (2 × 2 *μ*m^2^). For mechanical characterization, 65,536 measurements (256 × 256 independent force curves) were obtained for each image to yield the mean elastic modulus. All AFM raw files were processed with the Bruker NanoScope Analysis version 1.5. To identify the internal elastic lamellae (IEL) and external elastic lamellae (EEL) for localized measurement, the optical microscopy integrated with the AFM setup was utilized (Figure 1). Three random localized areas were probed in the media of each tissue section. Two sections were studied for each patient. Thus, there were six measurements of nanomechanical properties for each patient.

### 2.3. PeakForce QNM in Fluid Conditions

With the same AFM system, testing in fluid conditions was possible in a fluid cell (MTFEML, Bruker). A Bruker ScanAsyst-Fluid probe with an intentionally blunt tip (nominal tip radius = 20 nm) and extremely low spring constant (nominal spring constant = 0.7 Nm^−1^, resonant frequency = 150 kHz) was applied to image the delicate hydrated tissue sections in distilled water. The tip radius and spring constant of the probe were calibrated using the same procedure as used in ambient conditions [1]. Following this, a polydimethylsiloxane (PDMS) sample was used as the reference sample for modulus measurement calibration. The elastic modulus of PDMS was determined independently via nanoindentation (Nano Indenter G200 with a DCM II Head, KLA-Tencor, Milpitas, CA, USA) utilizing a 100 *μ*m flat punch (Synton-MDP Ltd, Nidau, Switzerland) before the calibration. The testing was conducted at 110 Hz using an oscillatory nanoindentation method [1]. The nanoindentation data yielded an average elastic modulus of 5.1 ± 0.36 MPa.

**Figure 2:**
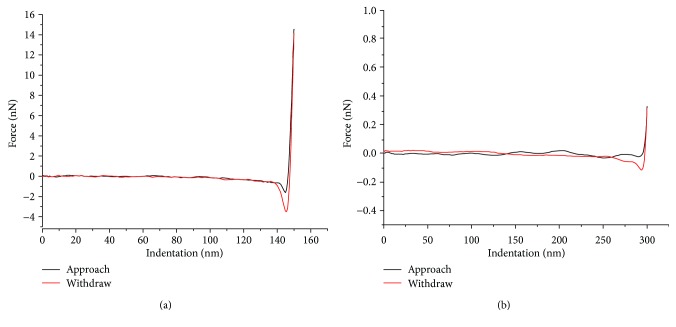
Examples of AFM force-indentation curves obtained from (a) PS1 and (b) PDMS reference samples in ambient and fluid conditions, respectively.

During liquid testing, sharp tips can drag across the hydrated soft tissue and cause damage to the tissue, thus causing errors in mechanical mapping. With the scan size set to 2 × 2 *μ*m with a slow scanning rate (less than 0.501 Hz), the mechanical mapping in liquid was found to be more reliable. Hence, for comparative purposes, imaging in ambient conditions was also kept at 2 × 2 *μ*m. The scan rate for the fluid tests was kept constant at 0.501 Hz. Further, due to the highly sensitive surface of the hydrated tissue samples, a higher scanning resolution was applied in the fluid conditions as compared with the measurements in ambient conditions. Thus, 384 samples/line were selected and 147,456 (384 × 384) independent force curves were obtained in each image to yield the mean elastic modulus. Example force-indentation curves are shown in Figure 2 for our reference samples in ambient and fluid conditions.

For each patient, two tissue sections were imaged with three random locations selected per tissue section. Thus, there were a total of six measurements per patient. However, samples from three patients (ID: 552 in low PWV, and 620 and 643 in high PWV; see Supporting Information, Table S1 in [1]) were unintentionally damaged during the hydration process and could not be studied. Thus, in total, seven patients in the low and high PWV groups were examined in the fluid condition.

### 2.4. Integration of Quantitative Proteomics, Nanomechanical Data, and Patient Metadata

Quantitative proteomics data for some of the patient cohort analyzed in this study were available from a larger cohort study published by Hansen et al. [9]. Full methodological data for these measurements can be found in their paper. The data available was for the 7 SLRP proteins that were found to be downregulated in the high PWV patients, namely, lumican, mimecan, prolargin, asporin, podocan, decorin, and biglycan [9]. To integrate the nanomechanical data acquired in this study with both proteomics data and patient metadata, a multivariate transformation called principal component analysis (PCA) was used.

### 2.5. Statistical Methods

Patient characteristics were presented as mean ± SD, and all the bar charts were presented as mean ± SEM. All patient measurements were averaged prior to statistical analysis. Group differences were assessed via a suitable 2-sample independent test selected after appraisal of data normality and homoscedasticity. Differences between nanomechanical properties of hydrated and dehydrated media of the low and high PWV group were tested with the Mann-Whitney *U* test. Kolmogorov-Smirnov (K-S) tests were applied to assess the statistical significance in distribution of nanomechanical properties of hydrated and dehydrated media between the low and high PWV groups. Spearman's correlation coefficient was used to test relationships between the measured elastic modulus of hydrated and dehydrated media and the PWV as well as with SLRPs expression activity. Proteomics data were analyzed in junction with nanomechanical variables and quantitative metadata variables (i.e., age, BMI, cholesterol, and HbA1c) of all the patients via the unsupervised data transformation PCA. All statistical analyses were tested using OriginPro version 9 (OriginLab, Northampton, MA).

## 3. Results and Discussion

In this study, the nanomechanical properties of hydrated and dehydrated media were assessed to determine the utility of this AFM for testing hydrated tissues. Assessment of the IMA medial layer in the different conditions is also compared with the adventitia data and evaluated with correlation analysis to assess the best approach for determining arterial stiffness from IMA samples. Histological assessment can be found in [1].

### 3.1. Nanomechanical Properties of Dehydrated Media

**Figure 3:**
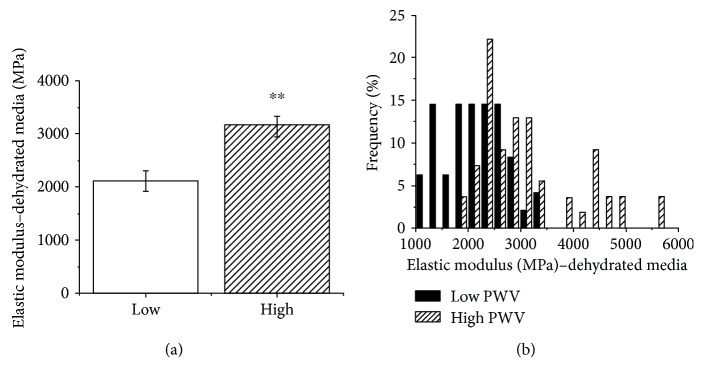
Nanomechanical properties of the dehydrated media in both groups in ambient condition. (a) Bar graph showing a significant difference in elastic modulus of patients as mean ± SEM (*n* = 8 patients in low PWV group; *n* = 9 patients in high PWV group). (b) Distribution of measured elastic modulus for both groups; significant differences were found between modulus distributions of two groups overall in the dehydrated media (Kolmogorov–Smirnov test, *p* < 0.0001).

In ambient conditions, patients with high PWV had a stiffer medial ultrastructure than that of the low PWV group (low PWV = 2116.2 ± 523.1 MPa; high PWV = 3163.6 ± 548.7 MPa, Mann-Whitney *U* test, *p* = 0.003) (Figure 3(a)). The elastic modulus ranged from 1325.5 to 2770.7 MPa and 2520.3 to 4030.0 MPa in the low and high PWV groups, respectively. Modulus distribution between these two groups was also found to be significantly different (Kolmogorov–Smirnov test, *p* < 0.0001) (Figure 3(b)).

### 3.2. Nanomechanical Properties of Hydrated Media

**Figure 4:**
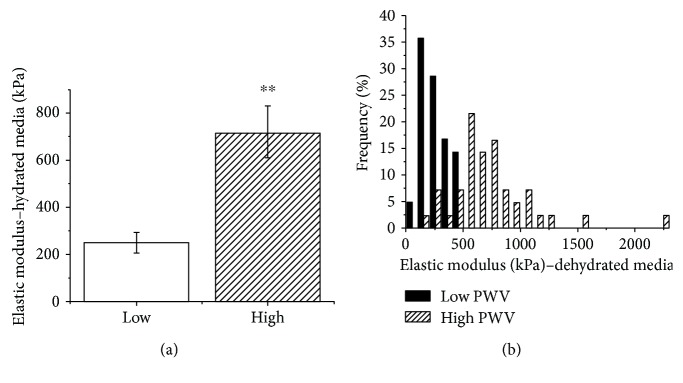
Nanomechanical properties of the hydrated media in the fluid condition in all available patients. (a) The elastic modulus of hydrated media in each patient (*n* = 7 patients per group, Mann–Whitney test, *p* = 0.005). (b) Modulus distribution of the medial layer in low and high PWV groups (*n* = 42 measurements per group, Kolmogorov–Smirnov test, *p* < 0.0001).

With the hydrated samples, similar trends were observed as those in ambient conditions; i.e., the elastic modulus was higher in the high PWV group. However, the trends were more pronounced in these conditions. In the high PWV group, the medial layer was approximately three times stiffer than that in the low PWV group (low PWV group = 250.6 ± 39.0 kPa; high PWV group = 721.7 ± 291.9 kPa, Mann–Whitney *U* test, *p* = 0.005) (Figure 4(a)). The measured elastic modulus of the patients ranged from 180.1 to 292.7 kPa and 286.6 to 1114.7 kPa in the low and high PWV groups, respectively. A significant difference was found between modulus distributions in the two groups (Kolmogorov–Smirnov test, *p* < 0.0001) (Figure 4(b)).

The mean elastic modulus was found to be 188% larger in the high PWV group as compared to the low PWV group. In comparison, an increase of 52% was observed when the tissues were dehydrated. The absolute values differed vastly in the two conditions with the elastic modulus being around three times higher when the tissue was tested dehydrated (Table 1). These data highlight the importance of water content in governing the mechanical properties of soft biological tissues.

The distribution of data was further assessed by examining the skewness and kurtosis as shown in Table 1. The low PWV group had a negative skewness in both hydration conditions whereas the high PWV group had a positive skewness. The kurtosis parameter was negative and approximately equivalent for the dehydrated media in both the low and high PWV groups. However, for the hydrated media, the kurtosis parameter was positive in the low PWV group and closer to zero in the high PWV group. Thus, in hydrated conditions, the tails in the elastic modulus distributions are more pronounced.

**Figure 5:**
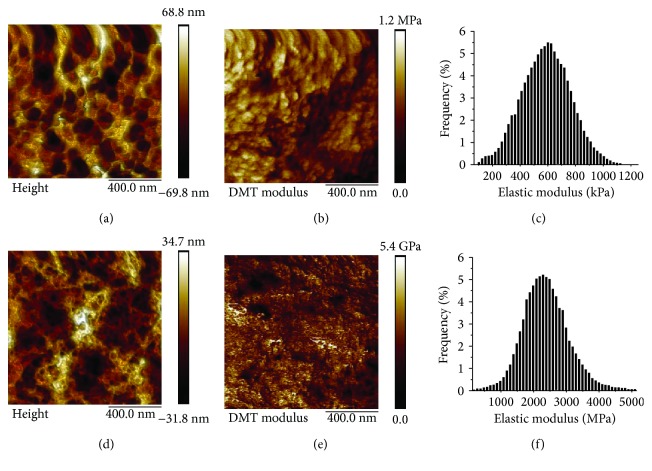
Example images showing the hydrated media (a) topography, (b) DMT modulus map, (c) histogram showing the elastic modulus distribution and for the dehydrated media, (d) topography, (e) DMT modulus map, and (f) histogram showing the elastic modulus distribution and for the dehydrated media mapping of topography. All images are for patient 627.

Figure 5 shows example AFM images for the medial layer in both dehydrated and hydrated conditions. The IMA is a transition artery, and in many patients, it is not classified as an elastic artery and hence does not have any elastic lamellae present [1]. Hence, the AFM images show an amorphous-type structure with little evidence of any fibrillar structure. This is more pronounced in the dehydrated images where the topographical variation is less pronounced (Figures 5(d) and 5(e)).

Although there are no other studies in the literature which have reported the elastic modulus of the human IMA medial layer, we can compare our values with other relevant published studies. Sicard et al. [18] reported values for human pulmonary arteries measured with AFM using a sharp pyramidal tip as being 67.66 ± 122.26 kPa with a range of 4.24–804.00 kPa. Akhtar et al. [4] used a frequency-modulated AFM method for characterizing the medial layer of young and old sheep aorta and reported a geometric mean of 42.9 ± 2.26 kPa for young sheep and 113.9 ± 2.57 kPa for old sheep. Grant and Twigg [19] used AFM with a 10 *μ*m tip to characterize the adventitial layer of porcine pulmonary arteries and porcine aorta. For the pulmonary arteries, they reported a range of 2.3–1130 kPa with a mean of 88.9 kPa, and for the porcine aorta a range of 0.7–391 kPa with a mean of 15.8 kPa. Our mean values are higher than each of these studies but within an acceptable range. Further work is needed to determine whether the difference is due to the test methods or the type of artery. Grant and Twigg [19] highlighted that there was considerable heterogeneity within arteries which also explains the large range of values.

### 3.3. Comparison of the Dehydrated Medial Layer vs. Dehydrated Adventitial Layer

We have previously published data demonstrating the utility of the IMA adventitial layer as a potential target for arterial stiffening studies [1]. Here, we now compare the mechanical data of dehydrated media with the adventitial data for the same patients [1], as well as the trends when the data are averaged for both layers (media and adventitia). These data are shown in Table 2. Overall, the elastic modulus in each condition was significantly increased in the high PWV relative to the low PWV group (low PWV: *n* = 8 patients; high PWV: *n* = 9 patients). Although there was no statistical difference in the measured elastic modulus between the dehydrated media and adventitia in each group (Mann–Whitney *U* test, *p* > 0.05), the dehydrated media was slightly stiffer than the dehydrated adventitia in the high PWV group. In the high PWV group, the ultrastructural stiffness increased by 49.5% and 34.1% for the dehydrated media and adventitia, respectively. For the combined layer group, the elastic modulus increased by 41.7% in the high PWV group.

### 3.4. Correlation Analysis

To assess the relationships between nanomechanical properties (PWV) and SLRP expression activity, Spearman's rank-order correlation was used to calculate the correlation coefficient for the relationships to test their significance (Table 3).

Overall, as shown in Table 3, the nanomechanical properties of hydrated and dehydrated media were significantly and positively correlated with the patient's PWV value, which was similar to the dehydrated adventitia [1]. The elastic modulus of the combined dehydrated media and adventitia was also positively related to the PWV data. These findings suggest that all four methods can be utilized to reflect clinical arterial stiffness, i.e., dehydrated adventitia, dehydrated media, or hydrated media.

For correlations between SLRP expression and nanomechanical properties of different layers in all the available patients (*n* = 12 patients for both conditions) (Table 3), 4 of 7 SLRPs showed a significant and negative correlation with measured elastic modulus of hydrated media. This corresponds to our previous study [9] where 5 of 7 SLRPs (lumican, mimecan, prolargin, aspirin, and decorin) were downregulated in patients with high PWV.

SLRPs as a family of complex and diverse macromolecules in arterial wall play an essential role in atherosclerosis [20] and arterial ECM remodeling [21]. The quantitative proteomics data collected from our previous study [9] from the entire IMA tissue have now been compared with nanomechanical measurements for the media (dehydrated and hydrated) and the adventitia (dehydrated), as well as for the media and adventitia combined (dehydrated). The correlation analysis indicates that the nanomechanical properties of the hydrated media are more related to the expression activity of SLRPs than the dehydrated media. This further highlights the importance of testing the tissue in conditions that mimic the physiological environment as much as possible.

SLRPs are essential in the structural integrity and functionality of the arterial wall by modulating synthesis, assembly, and remodeling of arterial ECM components such as collagen, elastin, and SMCs. The role of SLRPs in arterial stiffening, as well as on regulating collagen fibril assembly and architecture, has been discussed in detail elsewhere [9], and their relationship to adventitial collagen fibril morphology and nanomechanics has recently been established [1]. Alongside controlling collagen fibrillogenesis and collagen organization, studies have revealed the importance of SLRPs in regulating proliferation and migration of SMCs, which are predominant in the media of muscular arteries. Accumulated lumican, which is expressed in the SMCs, is located in the fibrous thickened intima and media where it associates with intimal thickening and is capable of maintaining adventitial mechanical properties in patients with atherosclerosis [22]. Highly expressed mimecan found in human SMCs is shown to accelerate cell proliferation, migration, and death, thereby regulating atherosclerosis [23] and atherosclerotic plaques [24]. Biglycan promotes SMC proliferation and migration in arteriosclerotic lesions related to arterial repair and pathogenesis of vascular injury [25]. Upregulated decorin is discovered in calcified regions in human atherosclerotic lesions, where it accelerates SMC calcification [26]. These important roles of SLRPs in the medial layer help shed light on why the SLRP expression is highly correlated with the medial layer nanomechanical properties.

### 3.5. Principle Component Analysis (PCA)

**Figure 6:**
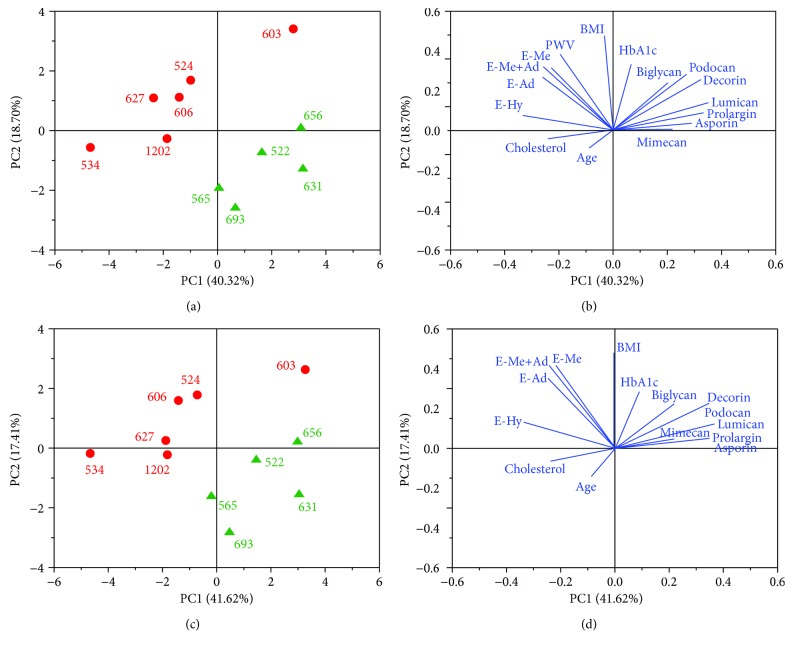
PCA of patient proteomics, quantitative metadata, and nanomechanical variables. (a) Score plot of the two first components. Each dot represents a patient. Patients colored by group (red - high PWV, green - low PWV). (b) Loading plot of (a) showing the variables that contribute the most of the structured observed in (a); PWV and elastic modulus of hydrated media (E-Hy), dehydrated media (E-Me), adventitia (E-Ad), and combined dehydrated media and adventitia (E-Me+Ad) are four of the most contributing variables to the separation between both groups. (c) Score plot of the two principal components of patient data without the PWV variable. Similar separation between both groups can be observed. (d) Loading plot of (c) shows that E-Hy, E-Me, E-Ad, and E-Me+Ad are four of the most contributing variables to the separation observed. E-Me and E-Ad were highly correlated. Of the SLRPs, mimecan presents the most contribution to the separation between groups observed.

The variables measured in this study were summarized and integrated with the SLRP data by PCA. The score plot for this transformation is shown in Figure 6(a) which clearly shows a difference between high and low PWV patients. The variables contributing the most to this separation are shown in the loading plot (Figure 6(b)) where, not surprisingly, it can be seen that PWV is one of the major contributors to the separation of the patients. Interestingly and in agreement with assessed data in this study, it is closely correlated with the elastic modulus of dehydrated layers. Furthermore, PCA on the data without the PWV variable shows similar separation between both high and low PWV patients and exhibits elastic modulus of the hydrated and dehydrated layers as three of the key variables to the separation (Figures 6(c) and 6(d)). The elastic modulus of hydrated media was closely related to age. In addition, the nanomechanical properties of dehydrated media and adventitia were closely correlated. Most SLRPs were closely grouped in the PCA analysis with some differences in mimecan and biglycan, which was similar to the PCA data of the adventitia.

## 4. Conclusions

This study probed the nanoscale stiffening-related ultrastructural changes in the tunica media of human IMAs in ambient and fluid conditions in patients with a high PWV. We show that PeakForce QNM can be used for mechanical property characterization of hydrated biological tissues. The study clearly demonstrates that AFM methods can be used as a tool for assessing arterial stiffness, with good agreement with expected trends based on *in vivo* measurements. The mechanical properties of the medial ultrastructure in both hydrated and dehydrated conditions correlated well with the carotid-femoral PWV. The AFM-derived nanomechanical properties were also correlated with SLRP expression, which is related to arterial stiffness. Based on the correlation tests, we suggest that the medial and adventitial layers are both suitable for AFM analysis to characterize nanoscale arterial stiffening. Overall, this study demonstrates the utility of AFM methods for arterial stiffening studies.

## Figures and Tables

**Table 1:** Mean, median, standard deviation (SD), standard error (SE), skewness, and kurtosis for the elastic modulus data are presented for the hydrated and dehydrated IMA media.

		*n*	Mean	Median	SD	SE	Skewness	Kurtosis
Hydrated media (kPa)	Low PWV	7	250.5	255.4	39.1	14.8	−0.9	0.8
	High PWV	7	721.7	649.6	291.9	110.3	0.2	−0.4
Dehydrated media (MPa)	Low PWV	8	2116.2	2142.3	523.1	184.9	−0.3	−1.2
	High PWV	9	3163.6	3044.5	548.7	182.9	0.6	−1.1

**Table 2:** Elastic modulus for the media, adventitia, and media + adventitia for the IMA tested when dehydrated. The Mann–Whitney *U* test was used to test the statistical difference between each layer of the low and high PWV groups.

	Media	Adventitia	Media and adventitia
Low PWV			
*n*	8	8	8
Mean (MPa)	2116.2	2159.3	2137.7
SD (MPa)	523.1	282.5	353.0
Coefficient of variation (%)	24.7	13.1	16.5
High PWV			
*n*	9	9	9
Mean (MPa)	3163.6	2895.2	3029.4
SD (MPa)	548.7	414.4	380.7
Coefficient of variation (%)	17.3	14.3	12.6
*p* value	0.003	0.002	0.002

**Table 3:** Spearman's rank-order correlation for assessing correlations of elastic modulus (E) of the different layers, PWV and SLRP expressions.

		Hydrated media	Dehydrated media	Dehydrated adventitia	Dehydrated media and adventitia
Number of patients	*n*	14	17	17	17
PWV	*r*	0.63	0.62	0.56	0.62
	*p*	0.017^∗^	0.008^∗∗^	0.02^∗^	0.009^∗∗^
Number of patients	*n*	12	12	12	12
Lumican	*r*	−0.71	−0.20	−0.50	−0.30
	*p*	0.01^∗∗^	0.54	0.10	0.34
Mimecan	*r*	−0.54	−0.02	−0.45	−0.22
	*p*	0.07	0.95	0.15	0.50
Prolargin	*r*	−0.72	−0.48	−0.52	−0.38
	*p*	0.008^∗∗^	0.12	0.09	0.23
Asporin	*r*	−0.62	−0.51	−0.39	−0.37
	*p*	0.033^∗^	0.09	0.21	0.24
Podocan	*r*	−0.48	−0.27	−0.34	−0.18
	*p*	0.11	0.39	0.28	0.57
Decorin	*r*	−0.63	−0.22	−0.34	−0.22
	*p*	0.028^∗^	0.48	0.29	0.48
Biglycan	*r*	−0.15	−0.12	−0.20	−0.07
	*p*	0.63	0.71	0.53	0.83

## Data Availability

The Excel data used to support the findings of this study are included within the supplementary information file.

## Supplementary Materials

Supplementary Materials The supplementary material is simply the raw data in an Excel spreadsheet in accordance with the journal's “Data Availability” policy.

## References

[1] Chang Z., Paoletti P., Barrett S. D. (2018). Nanomechanics and ultrastructure of the internal mammary artery adventitia in patients with low and high pulse wave velocity. *Acta Biomaterialia*.

[2] Palombo C., Kozakova M. (2016). Arterial stiffness, atherosclerosis and cardiovascular risk: pathophysiologic mechanisms and emerging clinical indications. *Vascular Pharmacology*.

[3] Graham H. K., Akhtar R., Kridiotis C. (2011). Localised micro-mechanical stiffening in the ageing aorta. *Mechanisms of Ageing and Development*.

[4] Akhtar R., Graham H. K., Derby B. (2016). Frequency-modulated atomic force microscopy localises viscoelastic remodelling in the ageing sheep aorta. *Journal of the Mechanical Behavior of Biomedical Materials*.

[5] Chung A. W., Booth A. D., Rose C., Thompson C. R., Levin A., van Breemen C. (2008). Increased matrix metalloproteinase 2 activity in the human internal mammary artery is associated with ageing, hypertension, diabetes and kidney dysfunction. *Journal of Vascular Research*.

[6] Engler A. J., Richert L., Wong J. Y., Picart C., Discher D. E. (2004). Surface probe measurements of the elasticity of sectioned tissue, thin gels and polyelectrolyte multilayer films: correlations between substrate stiffness and cell adhesion. *Surface Science*.

[7] Faarvang A.-S. A., Rørdam Preil S. A., Nielsen P. S., Beck H. C., Kristensen L. P., Rasmussen L. M. (2016). Smoking is associated with lower amounts of arterial type I collagen and decorin. *Atherosclerosis*.

[8] Preil S. A. R., Kristensen L. P., Beck H. C. (2015). Quantitative proteome analysis reveals increased content of basement membrane proteins in arteries from patients with type 2 diabetes mellitus and lower levels among metformin users. *Circulation: Cardiovascular Genetics*.

[9] Lyck Hansen M., Beck H. C., Irmukhamedov A., Jensen P. S., Olsen M. H., Rasmussen L. M. (2015). Proteome analysis of human arterial tissue discloses associations between the vascular content of small leucine-rich repeat proteoglycans and pulse wave velocity. *Arteriosclerosis, Thrombosis, and Vascular Biology*.

[10] Sweers K., Van Der Werf K., Bennink M., Subramaniam V. (2011). Nanomechanical properties of *α*-synuclein amyloid fibrils: a comparative study by nanoindentation, harmonic force microscopy, and Peakforce QNM. *Nanoscale Research Letters*.

[11] Eghiaian F., Rigato A., Scheuring S. (2015). Structural, mechanical, and dynamical variability of the actin cortex in living cells. *Biophysical Journal*.

[12] Lavanya Devi A. L., Nongthomba U., Bobji M. S. (2016). Quantitative characterization of adhesion and stiffness of corneal lens of *Drosophila melanogaster* using atomic force microscopy. *Journal of the Mechanical Behavior of Biomedical Materials*.

[13] Pletikapić G., Berquand A., Radić T. M., Svetličić V. (2012). Quantitative nanomechanical mapping of marine diatom in seawater using peak force tapping atomic force microscopy. *Journal of Phycology*.

[14] Adineh V. R., Liu B., Rajan R., Yan W., Fu J. (2015). Multidimensional characterisation of biomechanical structures by combining atomic force microscopy and focused ion beam: a study of the rat whisker. *Acta Biomaterialia*.

[15] Young T. J., Monclus M. A., Burnett T. L., Broughton W. R., Ogin S. L., Smith P. A. (2011). The use of the PeakForce^TM^ quantitative nanomechanical mapping AFM-based method for high-resolution Young’s modulus measurement of polymers. *Measurement Science and Technology*.

[16] Papi M., Paoletti P., Geraghty B., Akhtar R. (2014). Nanoscale characterization of the biomechanical properties of collagen fibrils in the sclera. *Applied Physics Letters*.

[17] The Reference Values for Arterial Stiffness' Collaboration (2010). Determinants of pulse wave velocity in healthy people and in the presence of cardiovascular risk factors: 'establishing normal and reference values'. *European Heart Journal*.

[18] Sicard D., Fredenburgh L. E., Tschumperlin D. J. (2017). Measured pulmonary arterial tissue stiffness is highly sensitive to AFM indenter dimensions. *Journal of the Mechanical Behavior of Biomedical Materials*.

[19] Grant C. A., Twigg P. C. (2012). Pseudostatic and dynamic nanomechanics of the tunica adventitia in elastic arteries using atomic force microscopy. *ACS Nano*.

[20] Talusan P., Bedri S., Yang S. (2005). Analysis of intimal proteoglycans in atherosclerosis-prone and atherosclerosis-resistant human arteries by mass spectrometry. *Molecular & Cellular Proteomics*.

[21] Barallobre-Barreiro J., Didangelos A., Schoendube F. A. (2012). Proteomics analysis of cardiac extracellular matrix remodeling in a porcine model of ischemia/reperfusion injury. *Circulation*.

[22] Onda M., Ishiwata T., Kawahara K., Wang R., Naito Z., Sugisaki Y. (2002). Expression of lumican in thickened intima and smooth muscle cells in human coronary atherosclerosis. *Experimental and Molecular Pathology*.

[23] Zhang H. J., Wang J., Liu H. F., Zhang X. N., Zhan M., Chen F. L. (2015). Overexpression of mimecan in human aortic smooth muscle cells inhibits cell proliferation and enhances apoptosis and migration. *Experimental and Therapeutic Medicine*.

[24] Fernández B., Kampmann A., Pipp F., Zimmermann R., Schaper W., Zahradka P., Wigle J., Pierce G. N. (2003). Osteoglycin expression and localization in rabbit tissues and atherosclerotic plaques. *Vascular Biochemistry*.

[25] Shimizu-Hirota R., Sasamura H., Kuroda M., Kobayashi E., Hayashi M., Saruta T. (2004). Extracellular matrix glycoprotein biglycan enhances vascular smooth muscle cell proliferation and migration. *Circulation Research*.

[26] Fischer J. W., Steitz S. A., Johnson P. Y. (2004). Decorin promotes aortic smooth muscle cell calcification and colocalizes to calcified regions in human atherosclerotic lesions. *Arteriosclerosis, Thrombosis, and Vascular Biology*.

